# Tyrosine Phosphorylation of NR2B Contributes to Chronic Migraines via Increased Expression of CGRP in Rats

**DOI:** 10.1155/2017/7203458

**Published:** 2017-03-14

**Authors:** Xiping Liang, Sha Wang, Guangcheng Qin, Jingmei Xie, Ge Tan, Jiying Zhou, Devin W. McBride, Lixue Chen

**Affiliations:** ^1^Chongqing Cancer Institute & Hospital & Cancer Center, Chongqing, China; ^2^Laboratory Research Center, The First Affiliated Hospital of Chongqing Medical University, Chongqing, China; ^3^Chongqing Key Laboratory of Neurology, Chongqing, China; ^4^Department of Physiology and Pharmacology, Loma Linda University, Loma Linda, CA, USA

## Abstract

Tyrosine phosphorylation of NR2B (NR2B-pTyr), a subunit of the N-methyl-D-aspartate (NMDA) receptor, has been reported to develop central sensitization and persistent pain in the spine, but its effect in chronic migraines has not been examined. We hypothesized that tyrosine phosphorylation of NR2B contributes to chronic migraines (CM) through calcitonin gene-related peptide (CGRP) in rats. Ninety-four male Sprague-Dawley rats were subjected to seven inflammatory soup (IS) injections. In a subset of animals, the time course and location of NR2B tyrosine phosphorylation were detected by western blot and immunofluorescence double staining. Another set of animals were given either genistein, vehicle, or genistein and recombinant CGRP. The mechanical threshold was measured, the expressions of NR2B-pTyr, NR2B, and CGRP were quantified using western blot, and nitric oxide (NO) was measured with the nitric acid reductase method. NR2B-pTyr expression, in neurons, peaked at 24 hours after CM. Genistein improved the mechanical threshold and reduced migraine attacks 24 and 72 hours after CM. Tyrosine phosphorylation of NR2B decreased the mechanical threshold and increased migraine attacks via upregulated CGRP expression in the rat model of CM. Thus, tyrosine phosphorylation of NR2B may be a potential therapeutic target for treatment of CM.

## 1. Introduction

Migraines are one of the most common neurological diseases and are defined as a recurrent, episodic, unilateral, often severe, and a mostly pulsating headache [1]. A subset of episodic migraineurs, however, develop chronic migraines, a condition characterized by headaches on P15 days/month for more than 3 months. It is estimated that the number of people who become burdened with chronic migraines increases by a rate of 2-3% per year [2, 3]. People with chronic migraines exhibit more disability and higher financial burden than those with episodic migraine. Since chronic migraine is frequently refractory to treatment and is extremely disabling, it is ranked as one of the most severe disabling diseases in industrialized countries [4].

While most studies are directed at understanding the pathophysiology of episodic migraine, the physiopathology of chronic migraines is not well understood. The current thinking is that chronic migraines are closely related to central sensitization which is induced by neurogenic inflammation. Hyperalgesia and frequency of migraine attacks are key features of chronic migraine [1, 4], and hyperglycemia and migraine attacks arise as a consequence of central sensitization pain pathways [5].

In the studies by Oshinsky and Gomonchareonsiri and Stucky et al., repeated inflammatory dural stimulation in rats was used to model repeated episodic activation of the trigeminovascular system seen in patients with hyperalgesia and frequency of headache attacks in chronic migraines [6, 7].

Convincing evidence demonstrates that the development of hyperalgesia and persistent pain involves activation of NMDA receptors in the spine [8, 9]. Phosphorylation of the NR1 and NR2A-D subunits is known to modulate NMDA receptor activity and affect synaptic transmission [9–14]. Among the signal transduction pathways for NMDA receptor activation, tyrosine phosphorylation of the NR2 subunits, particularly the NR2B subunit, plays a key role [8]. It has been shown that NR2B is one of the most important tyrosine phosphorylated proteins in the development of persistent pain induced by neurogenic inflammation. The enhanced tyrosine phosphorylation of NR2B may contribute to nociceptor activity-induced spinal plasticity and the development of central sensitization and persistent pain [15, 16]. Inhibition of NR2B tyrosine phosphorylation can prevent inflammation-induced spinal plasticity, the development of central sensitization, and persistent pain [8, 15].

There is strong evidence that calcitonin gene-related peptide (CGRP) is the key neuropeptide in migraines [17]. CGRP levels in the plasma, as well as in the cerebrospinal fluid, are increased during a migraine attack [18]. CGRP antagonists proved effective in acute migraine treatment [17, 18]. A recent study showed that there was an association between CGRP and NR2B in mechanical sensitization in rats [9]. However, there are few studies exploring either the role of NR2B tyrosine phosphorylation or the association of NR2B tyrosine phosphorylation and CGRP in chronic migraines.

The objective of the present study was to evaluate the effects of NR2B tyrosine phosphorylation of the trigeminal nucleus caudalis (TNC) on chronic migraine (CM) and the potential molecular mechanisms in rats.

## 2. Methods

### 2.1. Animals and the Animal Model

The experimental protocol was evaluated and approved by the Animal Care and Use Committee at Chongqing Medical University in China. Rats were housed at 22–24°C under a 12-hour light/dark cycle and allowed free access to water and food. The body weight for each rat was measured.

Male Sprague-Dawley rats weighing 260 to 320 g were subjected to craniotomy as described previously with some modification [6, 7]. Briefly, anesthesia was induced intraperitoneally with ketamine (80 mg/kg) followed by atropine (0.1 mg/kg). Rats were placed in a stereotaxic apparatus (ST-51603; Stoelting Co., Chicago, IL, USA) and fitted with a cranial chamber under anesthesia. A 1 mm wide craniotomy was performed over the transverse sinus along the midline, and a plastic cap with a stainless steel cannula was affixed to the bone using dental cement. The cannula's end opened into the dura, allowing the inflammatory soup (IS) or PBS to be in contact with the dura. After surgery, rats were kept at approximately 37°C on an electric heating blanket and were housed separately until complete recovery from anesthesia. The rats were allowed to recover for at least 1 week before IS or PBS stimulation. IS or PBS was slowly infused into the dura using a microinfusion pump through the dura cannula. This procedure was repeated three times per week for up to seven exposures.

### 2.2. Groups and Treatment

Rats were randomly assigned to the following groups: sham, CM, CM-vehicle, CM-genistein (an inhibitor of NR2B tyrosine phosphorylation), CM-genistein-CGRP, or vehicle. In the sham group, 20 *μ*L of PBS (pH 7.4) was slowly infused into the dura, as described above. In the chronic migraine group, 20 *μ*L of IS was infused. Following the IS infusion exposures in the CM-genistein and CM-genistein-CGRP groups, intracerebroventricular (ICV) injection of genistein (100 ng/g, 300 ng/g) or genistein and recombinant CGRP (re-CGRP) was performed with a microsyringe after the last IS stimulation, respectively [19]. For the CM-genistein-vehicle group, an equal amount of vehicle was injected.

### 2.3. Tactile Sensory Testing

Sensory testing was evaluated at 6, 12, 24, 48, and 72 hours after the last IS stimulation and at 24 and 72 hours after genistein treatment. Rats were acclimatized to the testing apparatus (20 × 20 cm) for 5–10 min during the daylight portion of their circadian cycle. We used the von Frey monofilament to test the von Frey pressure threshold on the midline of the forehead and the hind paw as described in previous studies [6, 8, 20]. The assigned force values ranged from 0 to 900 g. A positive response for the von Frey test was recorded when the rat vigorously stroked its face with the ipsilateral forepaw, quick recoil of the head away from the stimulus, or a paw withdrawal caused by stimulation. The response threshold is defined as the lowest force of two or more consecutive von Frey filaments which produces at least two responses to each filament.

### 2.4. Blood Sampling and NO Determination

Immediately at 24 hours after treatment, rats were euthanized. Blood (3 mL) was collected from the left ventricle using the cardiac puncture method [21]. Blood was collected into heparinized tubes and was centrifuged at 3000 RPM for 15 minutes. Plasma was collected and stored at −80°C. Nitric oxide (NO) kits were purchased from Nanjing Jiancheng Bioengineering Institute of China (Nanjing, China). The production of NO was analyzed by the nitric acid reductase method in which NO metabolites (nitrites and nitrates) were measured for NO quantification. All assays were carried out according to the manufacturer's instructions. The multiwall plates were read using a Benchmark microplate reader at 550 nm.

### 2.5. Immunofluorescence Staining

Rats were euthanized at 24 hours after genistein treatment for double immunofluorescence staining, as previously described [19], using a neuronal marker (anti-NeuN) (1 : 100, Millipore, Temecula, CA) and NR2B tyrosine phosphorylation (anti-NR2B-pTyr antibody) (1 : 100, Abcam). Brain sections in the trigeminal nucleus caudalis were incubated with a mixture of NeuN and NR2B-pTyr primary antibodies overnight at 4°C, followed by a mixture of secondary antibodies for 60 minutes at room temperature. Microphotographs were analyzed with a fluorescent microscope (Olympus OX51; Olympus Optical Co. Ltd., Tokyo, Japan). The number of positive cells was calculated as the mean of the numbers obtained from five pictures.

### 2.6. Western Blot

The samples for western blot were collected at 24 hours after treatment with genistein and re-CGRP. Proteins of the trigeminal nucleus caudalis were extracted by homogenizing in RIPA lysis buffer (sc-24948). Western blotting was performed as previously described [19]. Equal amounts of protein samples (50 *μ*g) were subjected to SDS-PAGE gel, electrophoresed, and transferred to a nitrocellulose membrane. Membranes were then blocked with a blocking buffer for 1 hour, followed by incubation overnight at 4°C with the primary antibodies: anti-NR2B (Santa Cruz Biotechnology, 1 : 3000 dilution), anti-NR2B-pTyr antibody (Santa Cruz Biotechnology, 1 : 3000 dilution), and anti-*β*-actin (mouse polyclonal) antibody (1 : 5,000) (Santa Cruz Biotechnology, CA), before washing three times with PBS. Membranes were incubated with goat anti-rabbit secondary antibody (Santa Cruz Biotechnology, 1 : 5000 dilutions) for 60 minutes at room temperature before washing three times with PBS. The immunoreactive bands were visualized with BeyoECL Plus (P1008, Beyotime Institute of Biotechnology) and quantified using a gel imaging system (ChemiDoc XRS, Bio-Rad, USA).

### 2.7. Statistical Analysis

The data were presented as means ± SEM. Parametric data was compared using one-way analysis of variance (ANOVA) followed by the Tukey method. Unpaired *t*-test was used to compare the difference between two groups. SPSS 13.0 was used for statistical analysis. A value of *P* < 0.05 was considered to represent statistical significance.

## 3. Results

### 3.1. NR2B Tyrosine Phosphorylation Was Increased in the Neurons of the TNC and Peaked at 24 to 48 Hours after the Last IS Stimulation in Chronic Migraine Rats

**Figure 1:**
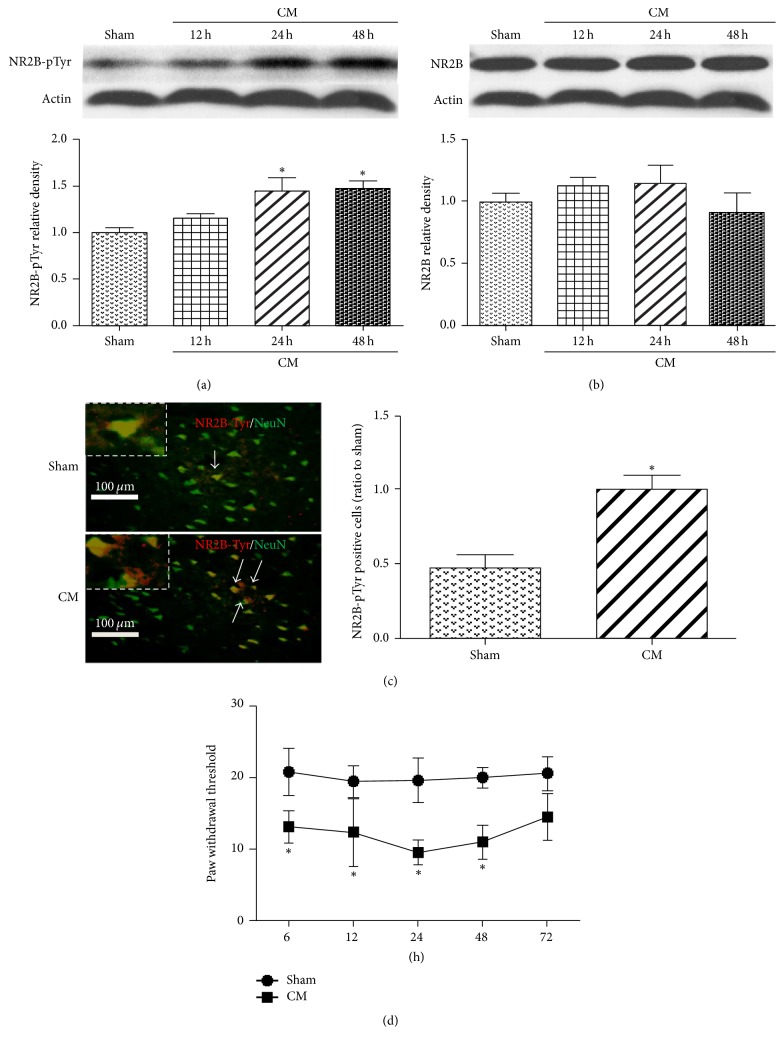
Time course and location of NR2B tyrosine phosphorylation (NR2B-pTyr) in TNC in CM rats. (a, b) Representative western blots of NR2B-pTyr and NR2B. NR2B-pTyr expression increased significantly after 24 and 48 hours and the level of NR2B was not changed significantly in CM rats. (c) Immunofluorescence double staining of NR2B-pTyr (red) and neuronal nuclei (NeuN, green) showed that the expression of NR2B-pTyr was increased and localized in TNC in CM. (d) Paw threshold was decreased in CM rats significantly. *n* = 8 per group for western blots and threshold; *n* = 5 per group for immunohistochemistry. ^*∗*^*P* < 0.05 versus sham. Bars: 100 *μ*m.

NR2B tyrosine phosphorylation (NR2B-pTyr) and NR2B expression at 12, 24, and 48 hours were measured by western blot in CM rats. Analysis showed that NR2B-pTyr began increasing at 12 hours and peaked at 24 hours which was sustained to 48 hours (^*∗*^*P* < 0.05 versus sham) (Figure 1(a)). The expression of NR2B was indistinguishable from sham (*P* > 0.05) (Figure 1(b)). To analyze the cellular location, double immunofluorescence staining was used. The representative pictures of the TNC sections stained with NR2B-pTyr and NeuN showed that there were more NR2B-pTyr positive cells colocalized with neurons compared to sham group (*P* < 0.05) (Figure 1(c)). The Tactile Sensory Test showed that mechanical threshold was decreased, accompanied by expression of NR2B-pTyr in CM rats. The mechanical threshold was decreased as early as 6 hours but reached a minimum at 24 hours which was sustained to 48 hours (^*∗*^*P* < 0.05 versus sham) (Figure 1(d)). The mechanical threshold returned to a level indistinguishable from sham by 72 hours.

### 3.2. Tyrosine Phosphorylation of NR2B Induced the Development of Chronic Migraine and Migraine Attacks

**Figure 2:**
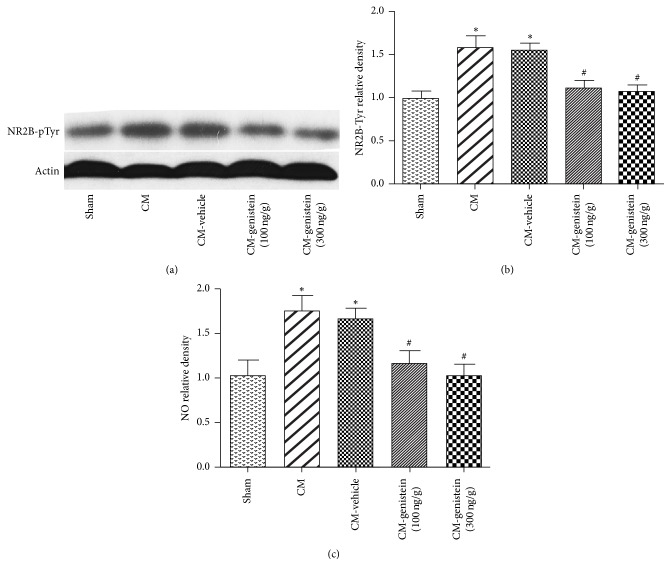
Tyrosine phosphorylation of NR2B induces the development of chronic migraine and migraine attacks. The expression of NR2B-pTyr by western blots (a, b) and the levels of NO (c) by the nitric acid reductase method were increased in chronic migraine. And the increased NR2B-pTyr and NO levels were reversed by both low and high dosage of genistein (inhibitor of NR2B tyrosine phosphorylation) in CM rats. ^*∗*^*P* < 0.05 versus sham; ^#^*P* < 0.05 versus CM-vehicle; *n* = 8 for each group.

To test whether tyrosine phosphorylation of NR2B affected chronic migraine in rats, male SD rats were intracerebroventricularly treated with genistein (an inhibitor of NR2B tyrosine phosphorylation) or vehicle. Western blots showed that NR2B tyrosine phosphorylation was increased in CM and vehicle groups which was reversed by both dosages of genistein at 24 hours after genistein administration (Figures 2(a) and 2(b); ^*∗*^*P* < 0.05 versus sham and ^#^*P* < 0.05 versus CM-vehicle). We used NO to test initiation and maintenance of migraine attacks by the nitric acid reductase method [22, 23]. Consistent with the level of NR2B tyrosine phosphorylation, NO content was increased in CM and CM-vehicle group (Figure 2(c), ^*∗*^*P* < 0.05 versus sham). The inhibitor of NR2B tyrosine phosphorylation reduced NO release caused by chronic migraine (Figure 2(c), ^#^*P* < 0.05 versus CM-vehicle). These data suggest that inhibition of NR2B tyrosine phosphorylation alleviated migraine attacks.

### 3.3. Inhibiting Tyrosine Phosphorylation of NR2B Ameliorated Hyperalgesia on the Mechanical Allodynia Induced by Chronic Migraine

**Figure 3:**
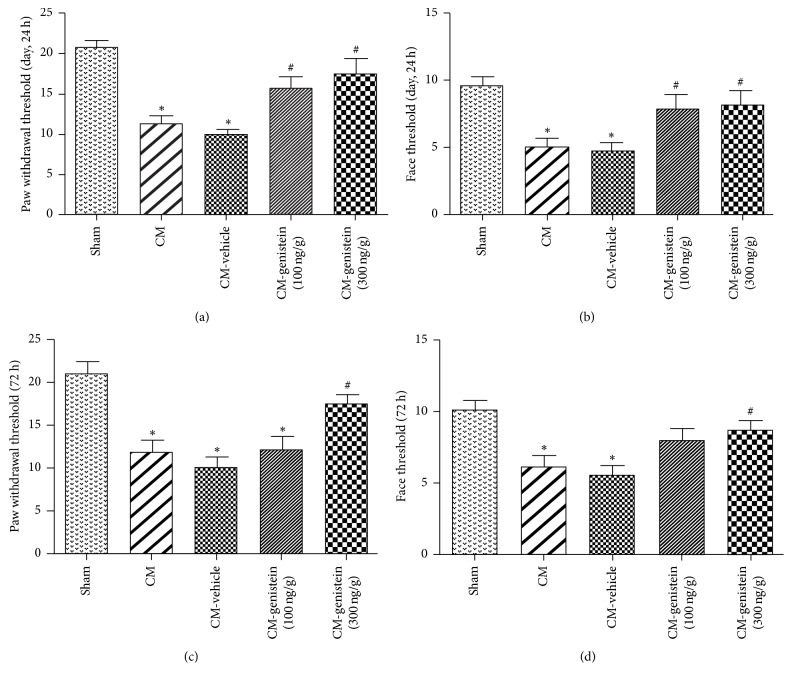
Inhibition of NR2B tyrosine phosphorylation led to protective function on allodynia at 24 hours and 72 hours after genistein treatment. Paw withdrawal (a, c) and face (b, d) threshold showed that high dosage of genistein led to both short- and long-term protective function on allodynia. ^*∗*^*P* < 0.05 versus sham; ^#^*P* < 0.05 versus CM-vehicle; *n* = 8 for each group.

The response to mechanical stimulation of each animal was recorded at 24 hours and 72 hours after genistein treatment. The threshold of paw withdrawal and face allodynia in the chronic migraine group and CM-vehicle group were significantly decreased compared with those in the sham group. Administration of genistein significantly improved both paw withdrawal and face allodynia induced by chronic migraine at 24 hours after genistein treatment (Figures 3(a) and 3(b), *P* < 0.05). To confirm a continued effect of genistein treatment on chronic migraine in rats, we measured the mechanical threshold at 72 hours after genistein treatment. Consistent with the result at 24 hours, the threshold was decreased in chronic migraine group and CM-vehicle group (Figures 3(c) and 3(d), ^*∗*^*P* < 0.05 versus sham), and the pain hypersensitivity was reversed in the high dosage of CM-genistein group significantly (Figure 3(d), ^#^*P* < 0.05 versus CM-vehicle). NR2B tyrosine phosphorylation induced allodynia in CM rats and its inhibitor led to protection from allodynia in chronic migraine.

### 3.4. Tyrosine Phosphorylation of NR2B Was Involved in Chronic Migraine through CGRP

**Figure 4:**
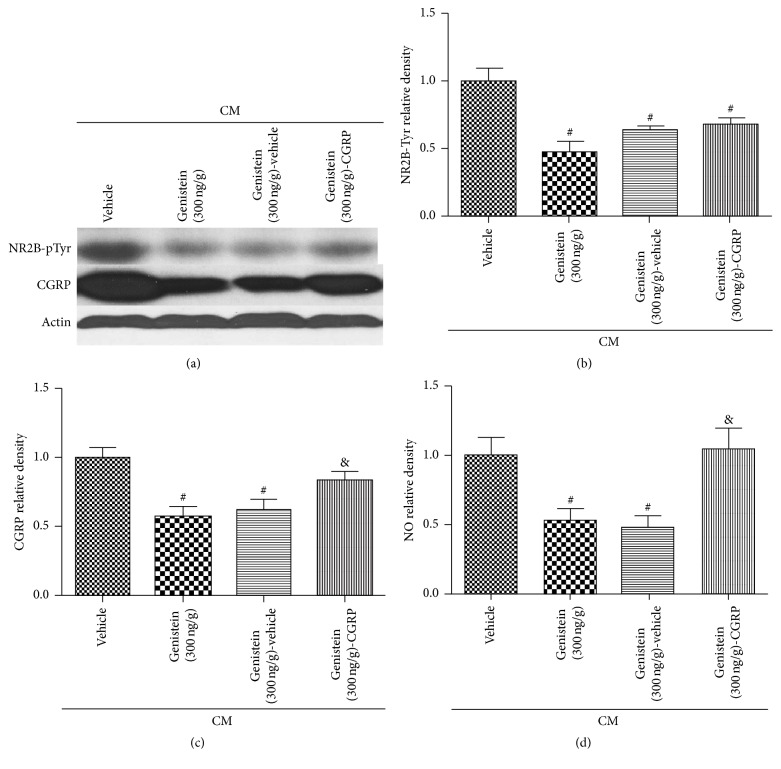
Tyrosine phosphorylation of NR2B took part in chronic migraine through CGRP in rats after 24 hours of treatment. Western blots of NR2B-pTyr and CGRP showed that treatment with genistein decreased expression of CGRP (c) and NO release (d). Treatment of recombinant CGRP increased the expression of CGRP (c) and NO release (d) but did not improve NR2B-pTyr levels. The level of NO was tested by the nitric acid reductase method. *n* = 8 for each group. ^#^*P* < 0.05 versus CM-vehicle; ^&^*P* < 0.05 versus CM-genistein (300 ng/g)-vehicle.

To test how tyrosine phosphorylation of NR2B affected chronic migraine in rats, we administered recombinant CGRP intracerebroventricularly and treated the rats with genistein. Western blot showed lower NR2B-pTyr and CGRP expression in CM-genistein and CM-genistein-vehicle groups compared with that of the CM-vehicle group (Figures 4(a), 4(b), and 4(c), ^#^*P* < 0.05). Genistein inhibited NO release in CM-genistein group and CM-genistein-vehicle group compared with CM-vehicle group (Figure 4(d), ^#^*P* < 0.05). Re-CGRP dramatically increased CGRP and NO levels (Figures 4(c) and 4(d), ^&^*P* < 0.05 versus CM-genistein-vehicle) but did not have any effect on the expression of NR2B-pTyr (Figure 4(b), ^&^*P* < 0.05 compared with CM-genistein-vehicle group). The results of western blot and NO levels showed that genistein reduces CGRP expression and migraine attacks, and administration of CGRP has no significant effect on NR2B-pTyr. The data supports the notion that tyrosine phosphorylation of NR2B plays a role in chronic migraine through CGRP.

## 4. Discussion

Recurrent migraine attacks and hyperalgesia on mechanical allodynia have been suggested to be an important characteristic of chronic migraines [1, 4]. Targeting and preventing migraine attacks and allodynia are logical therapeutic goals for chronic migraines. In this study, we provide the first evidence that suppressed tyrosine phosphorylation of NR2B ameliorates migraine attacks and hyperalgesia induced by chronic migraines. We also found that tyrosine phosphorylation of NR2B in the TNC was involved in chronic migraines, which was largely associated with its abilities to modulate the expression of CGRP. These observations suggest that suppressed NR2B tyrosine phosphorylation may be a potential therapeutic target for chronic migraines.

NR2B, a well-known subunit of the NMDA receptor, modulates the activation of the NMDA receptor and affects synaptic transmission [14, 24]. The NR2B subunit is the most important tyrosine phosphorylated protein in the brain, and it plays an important role in the phosphorylation of the NMDA receptor. Tyrosine phosphorylation of the NR2B subunit has been associated with the development of persistent pain. It was also connected with activity-induced spinal plasticity and the development of central sensitization in inflammatory pain. Moreover, expression of NR2B-pTyr was found in inflammatory pain in the spine and suppression of NR2B-pTyr ameliorates hyperalgesia [14, 16].

However, little was known about NR2B-pTyr's function in chronic migraines. We found that tyrosine phosphorylated NR2B was expressed in the TNC of chronic migraine rats, and there was a reduction of the mechanical threshold consistently. We further confirmed that tyrosine phosphorylated NR2B is located in neurons of the TNC, and administration of NR2B tyrosine phosphorylation inhibitor, genistein, decreases migraine attacks and leads to relief of hyperalgesia.

Calcitonin gene-related peptide (CGRP) is expressed in the trigeminal ganglia neurons which transmit nociceptive signals from head and face to the central nervous system [25]. Several lines of evidence support a central role of CGRP in migraine pathology [26–28]. Plasma CGRP levels, as well as CGRP cerebrospinal fluid levels, are increased during a migraine attack [18]. NO, a mark of initiation and maintenance of migraine attacks, contributes to the development of migraines. In this study, we found that CGRP and migraine attacks were increased in CM rats and that administration of an inhibitor of NR2B tyrosine phosphorylation reverses these observations.

How does NR2B tyrosine phosphorylation contribute to migraines in rats? Previous research has shown an association between the levels of the NMDA receptor and CGRP [9]. In our study, to ascertain NR2B tyrosine phosphorylation's function, we first examined the localization and time course of NR2B tyrosine phosphorylation in chronic migraine rats. NR2B tyrosine phosphorylation was found to be primarily expressed by neurons and was increased at 24 hours to 48 hours after the last IS injection, indicating that locally produced NR2B tyrosine phosphorylation may play an important role in chronic migraine. We also found that hyperalgesia and increased CGRP, as well as migraine attacks, were found in chronic migraine rats. Administration of an NR2B-pTyr inhibitor reversed the effects on hyperalgesia, migraine attacks, and CGRP expression. Delivery of recombinant CGRP increased CGRP levels and migraine attacks but did not increase the expression NR2B tyrosine phosphorylation in chronic migraine rats.

In conclusion, our findings indicate that NR2B tyrosine phosphorylation may contribute to chronic migraines in rats. Inhibition of NR2B tyrosine phosphorylation has a protective effect on the threshold dysfunction and migraine attacks via increased CGRP expression. This study provides new information on the function of NR2B tyrosine phosphorylation in chronic migraines and identifies NR2B as a novel candidate for the treatment of chronic migraines in patients.
